# Obstetrics, Gynecology, and Abdominal Surgery

**Published:** 1900-07

**Authors:** William Warren Potter

**Affiliations:** Buffalo, N. Y.


### Progress in Medical Science.

### OBSTETRICS, GYNECOLOGY, AND ABDOMINAL SURGERY.

By WILLIAM WARREN POTTER, M. D., Buffalo, N. Y.

### CAN ERGOT CAUSE RUPTURE OF THE UTERUS?

BONG (*Monatsheft für Geburtshülfe und Gynäkologie*, June, 1899, p. 900,) in a meeting of the Cologne Obstetrical and Gynecological Society, held February 10, 1899, discussed a case of uterine rupture in a multipara, to whom a midwife had administered a drachm of ergot within a short time. The pains became much stronger for a while, and then suddenly ceased with slight hemorrhage into the vagina. Bong was summoned, and found the fetal head in the pelvic inlet with the os dilated as large as a silver dollar. No fetal heart sounds. He made a diagnosis of atony and used expectant measures. On the next afternoon the patient collapsed. Examination revealed a right-sided uterine tear. Delivery was accomplished by turning, and manual extraction. The autopsy showed much blood in the peritoneal cavity. Bong was of the opinion that the ergot had produced uterine tetanus, and that the locality of least resistance had ruptured. The head was in the second vertex position, and the tear occurred in the right side of the lower segment. Ergot is doubtless at the bottom of many a case of so-called spontaneous rupture of the uterus at the hands of midwives. In the discussion Frank related a parallel case, and agreed with Bong in attributing the blame to ergot.

---

### UTERINE INERTIA.

DORSETT, (*Denver Medical Times*,) has used phosphate of strychnine (grain 1.100, gradually increased to grain 1.25) with very satisfactory results during the gestation period of anemic and debilitated women. In addition to the local action upon the uterine musculature, it promotes assimilation, relieves constipation and prevents the often observed chilliness or rigors after labor. Under this treatment he has not found it necessary to employ ergot for the past five years in his obstetric practice.

### THE EARLY SYMPTOMS OF PUERPERAL INFECTION.

Ferré (*L'Obstétrique*, ii, No. 5, p. 425,) points out that the general notion of the sudden onset of marked symptoms of puerperal infection after a longer and shorter period of silent incubation is inexact. Even in the period of incubation, important, although attenuated, symptoms may be present, and their recognition will greatly conduce to successful treatment. These early symptoms are: slight elevations of temperature occurring once or twice daily, and usually in the evening; a pulse rate of 80 or more, especially if in the morning, when the temperature is not yet raised; relative or absolute insomnia, which is a very important indication of serious infection and requires careful inquiry; headache, at first intermittent and slight, usually always in association with the other symptoms mentioned; sometimes diminution or suppression of the lochial discharge, although as a rule this is a later manifestation; and, finally, vague impressions of cold, but not usually a distinct rigor. The later symptoms, such as marked rigors, high temperature, local pain, etc., are well known; it is to the recognition of the early symptoms that we must trust for the successful treatment of such cases.

---

### PRURITUS VULVÆ.

This annoying and so often obstinate disturbance justifies frequent reference in journals. Herman (*British Med. Jour.*) makes the following classification which is most excellent:

1. Adventitious, due to dirt, pediculi, worms, or pessaries.

2. Skin diseases—eczema, herpes, or furuncle, follicular urticaria and diabetic dermatitis.

3. Irritating discharges, such as gonorrhea, cancer, senile endometritis; also cases in which no visible discharge is apparent.

4. Venous congestion, due to heart, liver and lung diseases.

5. Nervous affections.

For each division the following treatment is recommended:

1. White precipitate ointment for pediculi. For the other causes, absolute cleanliness and changing of the material of pessaries.

2. For eczema, usually affecting fat, elderly women and those pregnant, when due to pruritic organisms, warm hip baths, with liquor carboni detergens added, and the parts powdered with boric acid. When due to diabetes, general treatment. Herpes zoster did not respond to treatment. For follicular pruritus it is recommended to squeeze out the contents of follicles and apply corrosive sublimate, 1 to 2000.

3. Antiseptic and sedative douches, and sedative dusting powders on the vulva, as a saturated solution of borax and solution of boric acid. In case of failure with these try 1 to 7 solution of carbolic acid.

4. The same local treatment as for class two with general constitutional treatment.

5. Pruritus, when occurring in aged women, is frequently a symptom of degenerate changes, and treatment usually fails.

---

### THE EARLY DIAGNOSIS OF UTERINE CANCER.

WIENER, (*Amer. Jour. Obs. and Diseases of Women and Children*, December, 1899,) says: The early diagnosis of uterine malignant disease is one of the most difficult and responsible which the physician is called upon to make. Every error in diagnosis costs a human life; every delay endangers one. It is not only dangerous, but impossible, to make the diagnosis from symptoms alone, as there are no symptoms that are pathognomonic of cancer of the uterus. Pain seldom occurs in the early stages, and the discharge if present, is due to the pre-existing endometritis, and not to the new growth. Hemorrhage should always arouse suspicion, but in an organ which is subject to physiological hemorrhages the symptom is often overlooked, and especially when occurring at the menopause.

Malignant uterine disease is too apt to be looked for in advanced age only, while, in fact, statistics demonstrate conclusively that the majority of these cases are found before the menopause, and quite a number during the earlier years of sexual activity; fewer in old age. If pre-existing pregnancies influence the development of cancer it is indirectly, in that they give rise to endometritis. Endometritis is undoubtedly a very important etiological factor. It is present in almost all cases. The belief that uterine lacerations are predisposing causes is not now generally held. It develops in virgins and in nulliparæ, as well as in multiparæ. By inspection and palpation in the early stages, we cannot be sure of the structure of the new growth if that can be felt at all. By digital examination we can only find out which part of the uterus should be scraped most thoroughly. Microscopical examination of the uterine scrapings is the only accurate method of diagnosis. The whole interior of the uterus should be curetted most thoroughly, as approximately normal tissue is often found side by side with malignant degeneration. Dilate the cervical and uterine canals and use a sharp curette always, as a dull curette may not remove any part of the new growth if it originates as a hard nodule, or if it is some distance from the surface.

By early diagnosis alone can the number of operable cases be increased, and also the number of permanent cures. Jacobs, of Brussels, says that of seventy cases of uterine cancer in which he had removed the uterus only three were alive four years later. Thorn, of Magdeburg, reports that after five years less than 30 per cent. of the operated cases were alive. This is due to the fact that 70 per cent. of all cases are inoperable when first seen, and in many of the remaining 30 per cent. the disease has extended beyond the uterus. Bäcker, of Budapest, out of 705 cases, reports seventy, only 10 per cent. in which hysterectomy could be performed, and even of this small number very few remained radically cured. Malignant disease of the uterus must, by the scrapings, be distinguished from:

1. Endometritis, glandular and interstitial.
2. Necrotic myomata.
3. Polypi.
4 Placenta retenta.
5. Abortion.

---

CANCER OF THE UTERUS.

McMurtry, as a prophylaxis (*Amer. Prac. and News*, March 15, 1900,) in cancer says more operations ought to be done for lacerations of the cervix. A woman between forty and fifty years of age, who has borne children, ought invariably to be examined, and, when the physician finds she has a laceration of the cervix, a deep ulceration in the neck of the uterus, it ought to be repaired. Here is the greatest field of usefulness there is in connection with this disease. It is a very simple operation, it has no mortality, and it brings about a great deal of relief to the patient. All women who have the slightest disturbance of the menstrual function, or any hemorrhage from the uterus after the age of forty, ought to be very carefully examined with a view of finding an old laceration of the neck of the uterus, and, if such a condition be found, it ought to have immediate attention.

Sterile women and unmarried women who have never borne children do not, as a rule, have cancer of the uterus; multiparous women, who have borne a large family of children, are the ones especially prone to cancer of the uterus. The only way this can be explained is upon the hypothesis that there is some connection between laceration of the cervix and cancer; that there is something about the lacerated uterine neck which invites the morbid process.

